# Magnitude and determinant of healthcare-seeking behavior for childhood acute respiratory tract infections in Ethiopia: a cross-sectional study

**DOI:** 10.1186/s12887-023-04463-7

**Published:** 2024-01-03

**Authors:** Fantu Mamo Aragaw, Rediet Eristu Teklu, Meron Asmamaw Alemayehu, Nebiyu Mekonnen Derseh, Muluken Chanie Agimas, Daniel Alayu Shewaye, Atalay Liknaw Birhanie, Sintayehu Simie Tsega, Girum Shibeshi Argaw, Amensisa Hailu Tesfaye

**Affiliations:** 1 Department of Epidemiology and Biostatistics, Institute of Public Health, College of Medicine and Health Sciences, University of Gondar, Gondar, Ethiopia; 2 Department of Public Health, College of Health Science, Debre Markos University, Debre Markos, Ethiopia; 3 Department of Medical Nursing, School of Nursing, College of Medicine and Health Sciences, University of Gondar, Gondar, Ethiopia; 4 Department Of Nursing, College Of Medicine and Health Science, Jigjiga University, Jigjiga, Ethiopia; 5 Department of Environmental and Occupational Health and Safety, Institute of Public Health, College of Medicine and Health Sciences, University of Gondar, Gondar, Ethiopia

**Keywords:** Health care seeking behavior, Acute Respiratory Infection, Multilevel analysis, Ethiopia

## Abstract

**Background:**

Acute respiratory infections (ARIs) remain a major public health concern which become the leading cause of mortality and morbidity in children under the age of five. A large percentage of childhood deaths and complications can be avoided by seeking proper medical care. Therefore, this study aimed to assess the magnitude, and individual and community-level determinants of mothers’ healthcare-seeking behavior for their children under the age of five who had ARI symptoms in Ethiopia.

**Method:**

A secondary data analysis was conducted using the 2016 Ethiopian Demographic and Health Surveys(EDHS) with a total weighted sample of 643 under-five children who had ARI symptoms within two weeks of the survey. Due to the hierarchical nature of the EDHS data, a multi-level logistic regression model was used to identify the individual and community-level factors influencing mothers’ health care-seeking behavior for their children with ARI symptoms. In the multivariable multilevel analysis, those variables with a *p*-value < 0.05 were considered to be significant predictors of the outcome variable.

**Results:**

Healthcare-seeking behavior among mothers or caregivers for children with symptoms of ARIs was 32.61% (95% CI: 29.08–36.33%) in Ethiopia. The ICC in the null model indicated that about 55% of the total variability of treatment-seeking behavior was due to differences between clusters. Child aged > 24 months [AOR = 0.35; 0.19–0.63], having primary education [AOR = 3.25; 1.27–8.32], being media exposed [AOR = 2.49; 1.15, 5.38], female household head[AOR = 3.90; 1.35, 11.24], and delivery at health institution[AOR = 2.24; 1.00, 5.01] were significant predictors of health care seeking behavior of mother for their children with ARI symptoms.

**Conclusion:**

There is poor treatment-seeking behavior for children with symptoms of ARI in Ethiopia with significant community level variations. The multilevel logistic regression analysis showed that improving mothers’ education, women’s empowerment, facilitating institutional delivery and media accessibility are critical to promoting health-seeking behaviors among mothers or caregivers of under-five children with ARI symptoms. Hence, concerned bodies should design targeted interventions that increase mothers’ or caregivers’ treatment-seeking behavior for childhood ARI to reduce child morbidity and mortality.

## Introduction

The significant impact of child mortality in Ethiopia emphasizes the critical need for collaborative efforts to successfully address common health problems among children [1]. Acute respiratory infections (ARIs) continue to be a major public health issue, widely recognized as the leading cause of mortality and morbidity among children under the age of five [2]. ARIs are one of the most common causes of death in children around the world [3]. In Sub-Saharan Africa, acute respiratory infections are the major cause of mortality [4], and continue to kill more children under the age of five than other preventable infectious diseases [5, 6].

ARIs in children can have a significant impact especially if medical care is unavailable or not decided to seek [7]. Due to their immature immune systems, young childrens are more vulnerable to adverse outcomes [8]. One of the reasons for increased childhood morbidity and mortality is due to mothers’/caregivers’ lower healthcare-seeking behavior [9], and a large percentage of childhood deaths and complications can be avoided by seeking proper medical care [10]. Delays in seeking appropriate medical care for childhood illnesses are associated with a more negative outcome [11].

Recognizing symptoms was frequently regarded as a significant challenge in most studies of seeking care for ARI in children [12, 13]. Mothers and caregivers are frequently the first to detect symptoms of illness in their children [14]. Caregivers’ lack of awareness of the symptoms and danger signs may lead to delays or failure to seek treatment [4, 15]. Some evidence indicated that mothers did not possess the requisite knowledge to sufficiently recognize the significance of child health problems and seek professional healthcare advice [16].

Treatment for childhood illnesses is still difficult to obtain in countries with high child mortality rates [17–21]. Delays in seeking treatment for potentially fatal childhood diseases were linked to social beliefs and economic factors [22]. Health beliefs and prior experience with similar illnesses can also act as a barrier to seeking treatment for childhood illnesses [23].

Early detection and subsequent treatment of acute respiratory infections can help save lives [21, 24]. Incorporating health-seeking behaviour at appropriate health facilities into comprehensive management strategies is essential for effective prevention and treatment of childhood acute respiratory infections (ARIs) [25]. Most of previous studies were done using the ordinary/standard binary logistic regression which failed to consider the hierarchical nature of Demographic Health Survey (DHS) data, potentially introducing a bias to model estimates. Hence, we employed multivariable multilevel logistic analysis for such kind of hierarchical data to increase the statistical power and to get the appropriate estimation. This study aimed to assess the prevalence and to identify individual and community levels factors determining healthcare-seeking behaviour of mothers or caregivers for their children under five years with ARI symptoms in Ethiopia using nationally representative demographic and health survey data.

## Methods

### Data sources, sampling procedure, and populations

Secondary data analysis was carried out using the 2016 Ethiopian Demographic and Health Survey. The survey design employed a multi-staged stratified sampling technique. In the first stage, 645 EAs (202 in urban and 443 in rural areas) clusters or enumeration areas were chosen after stratifying each region into urban and rural areas. The second stage of selection involved choosing a fixed number of 28 households per cluster with an equal chance of systematic selection. Data were obtained from the DHS website: www.dhsprogram.com after a formal request. For this analysis, we used kids record data(KR file). A total weighted sample of 643 caregivers/mothers of children under the age of five five with symptoms of ARI were included in this study.

### Variables of the study

The outcome of this study was seeking healthcare for children under the age of five who had ARI symptoms in the two weeks preceding the interview. Seeking medical care was defined as seeking treatment from any health facility, excluding treatment from a traditional practitioner, by their mother/caregiver in the two weeks preceding the survey, and coded as “1”, otherwise “0” [26, 27].

The individual-level independent variables including in this study were, the age of the child, sex of the child, the age of the mother, the mother’s and partner’s education level, working status of the mother, household head, wealth index, place of delivery,sex of the child, covered by health insurance, and media exposure. The wealth index is a composite variable constructed using principal components analysis and categorized as “poorest,” “poorer,” “middle,” “richer,” and “richest” in the DHS dataset. For this study, we classified it into three categories: “poor” (including poorer and poorest), “middle,” and “rich” (including richer and richest). Media exposure status is created from the frequency of reading a newspaper or magazine, watching TV, and listening to the radio. If a woman answers yes to at least one question, she has been considered to have a media exposure.

Community-level factors included were place of residence(rural, urban), community-level poverty, distance to a health facility, community level of women’s education, and region. In the DHS survey, distance to a health facility was defined by asking respondents whether distance is a significant barrier to receiving when they are ill, and they describe it as a “big problem” or “not a big problem. Since the data were not normally distributed, community poverty and community level womens eduation were classified as high or low, by using the median value as the classification cut-off point. The poverty level in a community was categorized as high if the proportion of household in the two lowest wealth quintiles was greater than the median value, and low if the proportion was less than the median value [28]. The community level of women’s education was classified as high if the proportion of women with at least a primary level of education was greater than the median value, and low if it was less than the median value [29].

### Data processing and analysis

Before any statistical analysis, the data were weighted using sampling weight, primary sampling unit, and strata to restore the survey’s representativeness and to account for the sampling design and obtain reliable statistical estimates. The statistical software STATA version 14 were used for the analysis. We performed a multilevel logistic regression with a random effect at the cluster level, accounting for the clustered nature of the data as well as within and between community variations, and assuming that each community has a different intercept (β0) and fixed coefficient (β) [30]. Variables with p-value < 0.2 in the bi-variable analysis for both individual and community-level factors were fitted in the multivariable model. The results of the multilevel multivariable logistic regression model were reported in terms of adjusted odds ratios (AOR) and corresponding 95% confidence intervals (95% CI), with statistical significance determined at a level of p < 0.05. Four models were fitted in this multilevel analysis. The first was the null model containing no independent variables which were used to check the variability of healthcare-seeking behavior for ARI in the community. The second (model I) hierarchical models contain individual-level variables whereas the third (model II) contains community-level variables. In the fourth model (model III) both individual and community-level variables were considered in the analysis simultaneously. The goodness of the model fit was assessed using deviance information criteria (− 2×loglikelihood value).To test for multicollinearity among the independent variables, the Variance Inflation Factor (VIF) was used.

### Parameter estimation and model building

For the correlation between health care-seeking behavior for ARI and predictor variables, the fixed effects were used to calculate the odds ratio with a 95% confidence interval and a p-value of 0.05.

$$Log\;\left(\frac{\pi ij}{1-\pi ij}\right)={\mathrm\beta}_0+\mathrm\beta1\mathrm{xij}+\mathrm\beta2\mathrm{xij}+\mathrm\beta3\mathrm{xij}\dots\dots.\mathrm{uj}+\mathrm{eij}$$

Where πij = is the probability of seeking health care for ARI and 1-πij represents the probability of not seeking health care for ARI β1xij are individual and community level variables for the ith individual in group j, respectively.

The β’s are fixed coefficients indicating a unit increase in X can result in a β unit increase in the probability of healthcare-seeking behavior for ARI. While the β0 represents the intercept, which is the effect on healthcare-seeking behavior for ARI when all independent variables are absent. The uj represents the random effect, which is the community’s effect on health care-seeking behavior for ARI for the jth community, whereas eij represents random error at the individual level.

The random effects were measured with Intra-Class Correlation (ICC), and the median odds ratio (MOR),. The ICC demonstrates the differences between clusters in the healthcare-seeking behavior for ARI, and it is computed as ICC=$\frac{VA}{ VA+3.29}*100$, Where; VA =area-level variance [31–33]. The MOR indicates the central value of the odd ratio between the highest and the lowest risk regions when two clusters are chosen at random which is calculated as MOR=e0.95$\surd VA$, where VA = area level variance [30, 34].

### Ethical consideration

All methods were carried out following relevant guidelines of the Demographic and Health Surveys (DHS) program. All experimental protocols were approved by the Institutional Review Board (IRB) of University of Gondar. Informed consent was waived from the International Review Board of Demographic and Health Surveys (DHS) program data archivists after the consent paper was submitted to the DHS Program, a letter of permission to download the dataset for this study. The dataset was not shared or passed on to other bodies and was anonymized to maintain its confidentiality. All methods were carried out in accordance with relevant guidelines and regulations.

## Results

A total weighted sample of 643 women/caregivers of children with ARI symptoms were included in this study. Of these, around half of the children (51.15%) were females. Around two third of caregivers (68.21%) had no education. Half of the caregivers (50.41%) were less than 30 years of age. Around half of the children (51.06%) were less than 23 months of age, and the mean age of the children was 25.13 (SD = 15.97) months. In Ethiopia, healthcare seeking behavior for ARI is 32.61% (95% CI: 29.08-36.33%) ( Table 1).

**Table 1:** Weighted socio-demographic and health-related characteristics of the study participants in Ethiopia, 2016

Variables	Categories	Frequency	Percentage
Age of women	< 30	326	50.41
	> 30	321	49.59
Child age	< 24	330	51.06
	> 24	316	48.94
Marital status	Married	606	93.71
	Not married	40	6.29
Women education status	No education	441	68.21
	Primary	172	26.71
	Secondary and higher	32	5.08
Husband education status	No education	304	49.44
	Primary	271	44.16
	Secondary and higher	39	6.40
Current working status	Not working	343	53.09
	Working	303	46.91
ANC visit	No	208	32.16
	Yes	439	67.84
Place of delivery	Home	470	72.73
	Health institution	176	27.27
Sex of child	Male	316	48.85
	Female	331	51.15
Media exposure	No	442	68.29
	Yes	205	31.71
Household head	Male	580	89.74
	Female	66	10.26
Covered by health insurance	No	621	96.03
	Yes	25	3.97
Wealth index	Poor	294	45.42
	Middle	156	24.12
	Rich	197	30.47
Residence	Urban	42	6.53
	Rural	605	93.47
Community level poverty	Low	405	62.67
	High	241	37.33
Community level of women’s education	Low	298	46.06
	High	349	53.94
Distance to a health facility	Big problem	465	71.92
	Not a big problem	181	28.08
Region	larger central	624	96.42
	small peripherals	16	2.40
	metropolis	8	1.17

### Random effect analysis

The ICC in the null model indicated that about 55% of the total variability of treatment-seeking behavior was due to differences between clusters, with the remaining unexplained 45% attributable to individual variation. The MOR in the null model indicated that ,if we randomly choose an individual from two different clusters, those from a higher risk cluster had 5.20 times higher odds of having treatment-seeking behavior as compared to those individuals who come from the lower risk cluster. Also,the best-fitted model was the final model (model III) since it had the lowest deviance (598). All variables had VIF values less than 10, and the final model’s mean VIF value was 1.62, indicating the absence of multi-collinearity (Table 2).

**Table 2:** Parameters and model fit statistics for multilevel regression analysis models

**Random effect**				
	**Null model**	**Model I**	**Model II**	**Model III**
** VA**	4.07	6.14	4.01ssss	6.12
** ICC**	0.55	0.65	0.54	0.65
** MOR**	5.20	6.39	5.17	6.38
**Model comparisons**				
** Deviance**	718	605	700	598
** Mean VIF**	__	1.s24	1.38	1.62

*ICC *Inter cluster correlation coefficient, *MOR *Median odds ratio, *VA *Area level variance, *VIF *Variance inflation factor

## The fixed effect analysis result

In the final model, child age, educational status of the mother, media exposure,sex of household head, and place of delivery were associated with the treatment-seeking behavior of caregivers who had under-five children with ARIs symptoms.

This study showed that mothers who had children aged > 24 months were 65% (AOR = 0.35; 95% CI: 0.19–0.63) less likely to seek health care compared to those who had children aged < 24 months of age. Mothers who have primary education were 3.25 times (AOR = 3.25; 95% CI: 1.27–8.32) more likely to seek care for their children with symptoms of ARIs.

Mothers who had media exposure have 2.49 times (AOR = 2.49; 95% CI: 1.15–5.38) higher odds of seeking health care for their children with symptoms of ARIs than mothers with no media exposure. The odds of seeking health care for ARI were 3.90 times (AOR = 3.90; 95% CI: 1.35–11.24) higher among households that are headed by females as compared to women headed by males. Mothers who delivered their child at health facilities 2.24 times (AOR = 2.24; 95% CI: 1.00–5.01) were more likely to seek health care for their children with symptoms of ARIs compared to those mothers who were born at home(Table 3).

**Table 3:** Multilevel multivariable analysis of factors associated with mothers’ health care seeking behavior for ARI episodes in Ethiopia, 2016

Variables	Catsegories	Null model	Model I	Model II	Model III
			AOR [95% CI]	AOR [95% CI]	AOR [95% CI]
Age of women	<30		1.00		1.00
	>30		0.99 [0.50, 1.95]		1.05 [0.52, 2.09]
Child age	<24		1.00		1.00
	>24		0. 33 [0.18, 0.31]		0.35 [0.19, 0.63] ***
Women education status	No education		1.00		1.00
	Primary		2.24 [0.96, 5.21]		3.25 [1.27, 8.32]*
	Secondary and higher		1.86 [0.31, 11.24]		2.20 [0.31, 15.28]
Husband education status	No education		1.00		1.00
	Primary education		1.04 [0.49, 2.17]		1.01 [0.47, 2.17]
	Secondary & higher education		1.06 [0.28, 3.97]		0.95 [0.25, 3.62]
Current working status	Not working		1.00		1.00
	Working		1.49 [0.75, 2.95]		1.59 [0.78, 3.23]
Media Exposure	No		1.00		1.00
	Yes		2.49 [1.18, 5.27]		2.49 [1.15, 5.38]*
Household head	Male		1.00		1.00
	Female		4.22 [1.48, 11.95]		3.90 [1.35, 11.24]*
Covered by health insurance	No		1.00		1.00
	Yes		1.27 [0.31, 5.19]		1.27 [0.30, 5.25]
Place of delivery	Home		1.00		1.00
	Health institution		2.40 [1.11, 5.18]		2.24 [1.00, 5.01]*
Sex of child	Male		1.00		1.00
	Female		0.64 [0.36, 1.15]		0.63 [0.35, 1.15]
Wealth index	Poor		1.00		1.00
	Middle		0.98 [0.44, 2.22]		1.02 [0.41, 2.51]
	Rich		2.13 [0.98, 4.63]		2.02 [0.84, 4.87]
**Community level variables**					
Residence	Urban			6.01 [1.25, 28.77]	3.00 [0.37, 24.31]
	Rural			1.00	1.00
Community level poverty	Low			1.00	1.00
	High			0.56 [0.22, 1.42]	0.86 [0.24, 3.03]
Community level of women's education	Low			1.00	1.00
	High			1.18 [0.49, 2.80]	0.36 [0.11, 1.19]
Distance to a health facility	Big problem			1.00	1.00
	Not a big problem			0.94 [0.50, 1.74]	0.73 [0.33, 1.62]
Region	Small peripheral			1.00	1.00
f	Large central			0.29 [0.05, 1.60]	0.23 [0.03, 1.78]
	Metropolitans			1.11 [0.05, 23.44]	0.66 [0.01, 27.07]

*AOR *Adjusted odds ratio, *CI *Confidence interval.

* = *P*-value < 0.05, ** = *P* value < 0.01, *** = *P *value < 0.001

## Discussion

Seeking treatment from a healthcare setting for common childhood illnesses is extremely important in reducing child mortality.One-third of mothers or caregivers seek health care for their child with symptoms of ARIs which is lower than in a previous study done in Ethiopia [35]. Mothers with children older than 24 months were less likely to seek health care than mothers with children younger than 24 months. The finding is similar to a study done in Ethiopia [9] Indonesia [7], a study done in low and middle-income countries [36], and Bangladesh [3]. One possible explanation is that young children were perceived to be more sensitive and in need of immediate attention, and thus were more likely to be taken to health facilities [7].

Mothers who have primary education were more likely to seek care for their children with symptoms of ARIs than mothers who have no education. The findings are similar to those of a study conducted in Ethiopia [9], and a study done in low and middle-income countries [36].The reason for this could be that educated mothers are more aware of the etiology of diseases, illness symptoms, danger signs, and their consequences, which will increase the level of health care seeking for their children [37]. Furthermore, education is likely to increase female autonomy, allowing women to make more informed health-care decisions for their children [38].

Mothers who have been exposed to the media have a higher likelihood of engaging in health-seeking behavior for their children who have ARI symptoms than mothers who have not been exposed to the media. The result is similar to a study done in India [39], West Bengal [40], and Tanzania [41]. One possible explanation is that mass media exposes people to messages about healthcare management, which improves their health-seeking behavior. Evidence also suggests that exposure to the media is important for promoting healthy behaviors [41].

The odds of seeking health care for ARI were higher in female-headed households than in male-headed households. The finding is similar to a study done in sub-Saharan Africa [42], and Tanzania [41]. The reason for this could be that households with female heads do not have to face the challenge of obtaining permission to seek care, which is a common barrier for health care utilization among women and children [43]. Another possible explanation is that women who are heads of households have a tendency to use the available resources they control to prioritise health of their children, which increases their health-seeking behaviour [44].

Mothers who gave birth in a health facility were more likely to seek health care in the event of an ARI episode than mothers who gave birth at home. The finding is similar to a study done in Ethiopia [35], and Nigeria [45]. The possible reason might be that women who deliver at health facilities may have an opportunity for health promotion, which increases the likelihood of a subsequent visit to the health care facility [46, 47].

The study’s strengths included the use of a weighted nationally representative dataset and an advanced model that took the data’s hierarchical structure into account. As a limitation, the data did not include information about some predictor variables such as mothers or caregivers knowledge and perception regarding the illness since it was secondary data analysis. Also, only children who exhibited symptoms were included in our study, potentially leading to selection bias since mild symptoms may not recognized as symptomatic by their parents.

## Conclusion

There is poor treatment-seeking behavior for children with symptoms of ARI in Ethiopia with significant community level variations. The multilevel logistic regression analysis showed that improving mothers’ education, women’s empowerment, facilitating institutional delivery and media accessibility are critical to promoting health-seeking behaviors among mothers or caregivers of under-five children with ARI symptoms. Hence, concerned bodies should design targeted interventions that increase mothers’ or caregivers’ treatment-seeking behavior for childhood ARI to reduce child morbidity and mortality.

## Data Availability

The datasets used and/or analyzed during the current study are available from the corresponding author upon reasonable request.

## Abbreviations

AOR: Adjusted Odds Ratio
ARI: Acute respiratory infection
CI: Confidence Interval
COR: Crude odds ratio
DHS: Demographic and Health Survey
EAs: Enumeration Areas
EDHS: Ethiopian Demographic and Health Survey
ICC: Intra Class Correlation
MOR: Median Odd Ratio
PCV: Proportional Change in Variance
VA: Area level variance
VIF: Variance inflation factor
WHO: World Health Organization

## References

[1] Takele K, Zewotir T, Ndanguza D (2019). Risk factors of morbidity among children under age five in Ethiopia. BMC Public Health.

[2] Adesanya OA (2016). C.J.B.p.h. Chiao, *a multilevel analysis of lifestyle variations*. Symptoms of Acute Respiratory Infection among Young Children under Five in Nigeria.

[3] Sultana M (2019). Prevalence, determinants and health care-seeking behavior of childhood acute respiratory tract Infections in Bangladesh. PLoS One..

[4] Noordam AC (2017). Association between caregivers’ knowledge and care seeking behaviour for children with symptoms of Pneumonia in six sub. -Saharan Afr Ctries.

[5] Sadruddin S (2012). Househ Costs Treat Severe Pneumonia Pakistan.

[6] Shibata T (2014). Childhood acute Respiratory Infections and household environment in an eastern Indonesian urban setting. Int J Environ Res Public Health..

[7] Titaley CR (2020). Health care–seeking behavior of children with acute respiratory infections symptoms: analysis of the 2012 and 2017 Indonesia Demographic and Health Surveys. Asia Pac J Public Health.

[8] Liu L (2016). Levels and Causes of Mortality under age Five Years.

[9] Abegaz NT, Berhe H, Gebretekle G.B.J.B.p. (2019). Mothers/caregivers healthcare seeking behavior towards childhood Illness in selected health centers in Addis Ababa, Ethiopia: a facility-based cross-sectional study. BMC Pediatr..

[10] Geldsetzer P (2014). The recognition of and care seeking behaviour for childhood Illness in developing countries: a systematic review. PLoS One..

[11] Källander K (2008). Delayed care seeking for fatal Pneumonia in children aged under five years in Uganda: a case-series study. Bull World Health Organ..

[12] Nichter M, Nichter MJMa (1993). Acute Respiratory Illness: Popular Health Culture and Mother’s Knowledge in the Philippines.

[13] Zaman K (1997). Acute lower Respiratory Infections in rural Bangladeshi children: patterns of treatment and identification of barriers. Southeast Asian J Trop Med Public Health..

[14] Oyekale A (2015). Assessment of Malawian mothers’ Malaria knowledge, healthcare preferences and timeliness of seeking Fever treatments for children under five. Int J Environ Res Public Health..

[15] Ndu IK et al. Danger signs of childhood pneumonia: caregiver awareness and care seeking behavior in a developing country 2015. 2015. 10.1155/2015/167261 PMC4632178 26576161

[16] Fujino Y (2009). Improvement in mothers’ immediate care-seeking behaviors for children’s danger signs through a community-based intervention in Lusaka. Zambia. Tohoku J Exp Med..

[17] Colvin CJ (2013). Understanding careseeking for child illness in sub-Saharan Africa: a systematic review and conceptual framework based on qualitative research of household recognition and response to child diarrhoea, pneumonia and malaria. Soc Sci Med.

[18] Mosites EM (2014). Care-seeking and appropriate treatment for childhood acute respiratory illness: an analysis of demographic and health survey and multiple indicators cluster survey datasets for high-mortality countries. BMC Public Health..

[19] Herbert HK (2012). Care seeking for neonatal Illness in low-and middle-income countries: a systematic review. PLoS Med..

[20] Barros AJ (2012). Equity in maternal, newborn, and child health interventions in countdown to 2015: a retrospective review of survey data from 54 countries. Lancet..

[21] Noordam AC, et al. Care seeking behavior for children with suspected pneumonia in countries in sub-Saharan Africa with high pneumonia mortality. PLoS One. 2015;10(2):e0117919. 10.1371/journal.pone.0117919 PMC4338250 25706531

[22] Nonyane BA (2016). Factors associated with delay in care–seeking for fatal neonatal illness in the Sylhet district of Bangladesh: results from a verbal and social autopsy study. J Glob Health..

[23] Motsima TJEJoM, Sciences H. The Risk Factors Associated With Early Age At First Birth Amongst Angolan Women: Evidence From The 2015–2016 Angola Demographic And Health Survey 2020. 2(2).

[24] UNICEF W, W, UNICEF. Pneumonia: the forgotten killer of children. UNICEF/WHO 140. 2006.

[25] Gove S (1997). Integrated management of childhood illness by outpatient health workers: technical basis and overview. The WHO working group on guidelines for integrated management of the sick child. Bull World Health Organ..

[26] Croft TN, et al. Guide to DHS statistics, vol 645. Rockville: ICF; 2018.

[27] Chilot D (2022). Factors associated with healthcare-seeking behavior for symptomatic acute Respiratory Infection among children in East Africa: a cross-sectional study. BMC pediatrics..

[28] Liyew AM, Teshale AB (2020). Individual and community level factors associated with anemia among lactating mothers in Ethiopia using data from Ethiopian demographic and health survey, 2016; a multilevel analysis. BMC Public Health..

[29] Sisay D, et al. Spatial Distribution and Associated Factors of Institutional Delivery among Reproductive-Age Women in Ethiopia: The Case of Ethiopia Demographic and Health Survey. Obstet Gynecol Int. 2022;2022:1-12. 10.1155/2022/4480568 PMC9252845 35795329

[30] Merlo J (2005). A brief conceptual tutorial on multilevel analysis in social epidemiology: interpreting neighbourhood differences and the effect of neighbourhood characteristics on individual health. J Epidemiol Community Health..

[31] Austin PC (2017). J.J.S.i.m. Merlo. Intermediate and Advanced Topics in Multilevel Logistic Regression Analysis.

[32] Sommet N, Morselli D (2017). Keep calm and learn multilevel logistic modeling: A simplified three-step procedure using Stata, R, Mplus, and SPSS. International Review of Social Psychology..

[33] Weinmayr G (2017). Multilevel regression modelling to investigate variation in Disease prevalence across locations. Int J Epidemiol..

[34] Merlo J (2006). A brief conceptual tutorial of multilevel analysis in social epidemiology: using measures of clustering in multilevel logistic regression to investigate contextual phenomena. J Epidemiol Community Health..

[35] Geda NR (2021). Disparities in mothers’ healthcare seeking behavior for common childhood morbidities in Ethiopia: based on nationally representative data. BMC Health Serv Res..

[36] Bogler L, et al. Health-care seeking for childhood diseases by parental age in Western and Central Africa between 1995 and 2017: A descriptive analysis using DHS and MICS from 23 low- and middle-income countries. J Glob Health. 2021;11:13010. 10.7189/jogh.11.13010 PMC8397328 34484717

[37] Degefa G (2018). Determinants of delay in timely treatment seeking for Diarrheal Diseases among mothers with under-five children in central Ethiopia: a case control study. PLoS One..

[38] Chen E, Matthews KA, Boyce WTJ (2002). Socioeconomic differences in children’s health: how and why do these relationships change with age?. Psychol Bull..

[39] Chandwani H, Pandor JJEp (2015). Healthcare-seeking Behav Mothers Regarding Their Child Tribal Community Gujarat India.

[40] Ghosh N, et al. Factors affecting the healthcare-seeking behavior of mothers regarding their children in a rural community of Darjeeling district, West Bengal. Int J Med Public Health. 2013;3(1).

[41] Adinan J (2017). Individual and contextual factors associated with appropriate healthcare seeking behavior among febrile children in Tanzania. PLoS One..

[42] Akinyemi JO (2019). Household relationships and healthcare seeking behaviour for common childhood illnesses in sub-Saharan Africa: a cross-national mixed effects analysis. BMC health services research..

[43] Charles J (2008). The role of mothers in household health-seeking behavior and decision-making in childhood febrile Illness in Okurikang/Ikot Effiong Otop Community. Cross River State Nigeria.

[44] Richards E (2013). Going beyond the surface: gendered intra-household bargaining as a social determinant of child health and nutrition in low and middle income countries.

[45] Adeoti IG, Cavallaro FL (2022). Determinants of care-seeking behaviour for fever, acute respiratory infection and diarrhoea among children under five in Nigeria. Plos one..

[46] Lawn J, Kerber K. Opportunities for Africa’s Newborns: Practical data, policy and programmatic support for newborn care in Africa, Vol 32. Cape Town: Partnership for Maternal, Newborn and Child Health; 2006.

[47] Fekadu GA (2018). The effect of antenatalcare on use of institutional delivery service and postnatal care in Ethiopia: asystematic review and meta-analysis. BMC Health Serv Res.

